# Diet, gut microbiome, and cognition in neurodegeneration: a review and methodological framework

**DOI:** 10.3389/fnagi.2026.1771904

**Published:** 2026-03-04

**Authors:** Jacob Raber, Thomas J. Sharpton

**Affiliations:** 1 Departments of Behavioral Neuroscience, Neurology, and Radiation Medicine, Division of Neuroscience, ONPRC, Oregon Health and Science University, Portland, OR, United States; 2 Department of Microbiology and Statistics, Linus Pauling Institute, Oregon State University, Corvallis, OR, United States

**Keywords:** Alzheimer’s disease, cognition, diet questionnaire, gut microbiome, Parkinson’s disease

## Abstract

The gut microbiome influences brain function through the gut-brain axis via synthesis of neurotransmitters, production of metabolites affecting epithelial barrier integrity and immune modulation and signaling through the vagus nerve. In humans, microbiome diversity reflects healthy aging and predicts survival, while dysbiosis is increasingly implicated in neurodegenerative conditions including Alzheimer’s disease, Parkinson’s disease, multiple sclerosis, and ALS. Fecal transplant studies in germ-free mice demonstrate that microbiome alterations are sufficient to induce cognitive and neuropathological phenotypes, supporting causality in preclinical models. Genetic risk factors and environmental exposures affect both neurodegeneration risk and microbiome composition. In this review, we synthesize evidence from human cohorts and preclinical models on the gut-brain axis in cognitive health and disease. We then present a methodological framework for diet-microbiome-cognition research, addressing causal inference through mediation analysis, supervised approaches for deriving diet scores, validation strategies, and individual heterogeneity. This framework can guide development of microbiome-targeted dietary interventions to improve cognitive outcomes.

## 1 Introduction

### 1.1 The gut-brain axis and measures of cognition in humans and animal models

**Figure 1:**
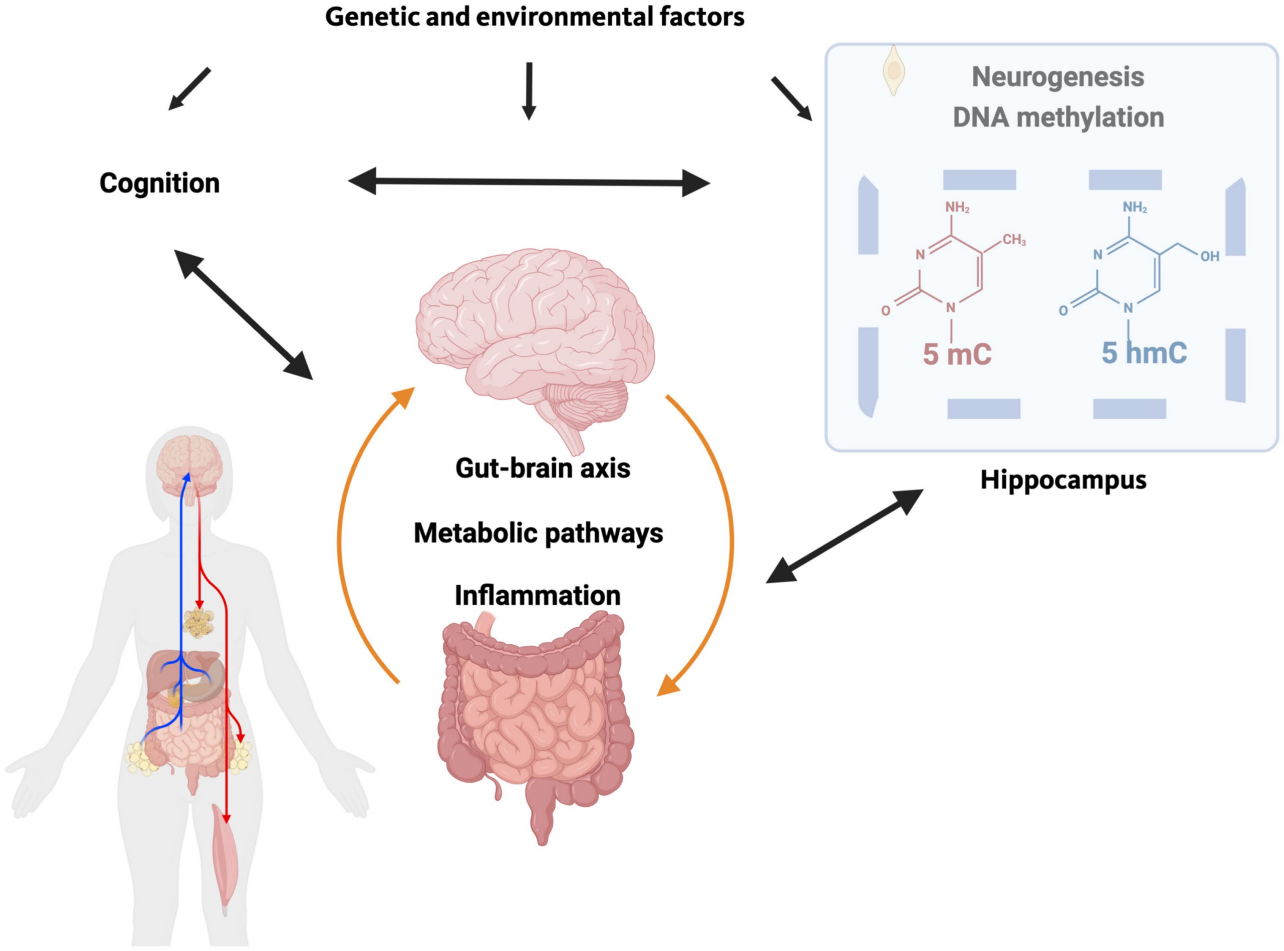
The gut-brain axis involves bi-directional communication. Inflammation in the gut and the brain and metabolic pathways play a role in this communication. Genetic and environmental factors affecting cognitive performance in cognitive health and cognitive injury in neurodegenerative conditions might affect the gut-brain axis. Alterations in epigenetic pathways and neurogenesis in the hippocampus, a brain area involved in cognitive performance and susceptible to injury, might mediate some of the effects of the gut-brain axis in health and neurological diseases.

The gut brain axis involves bi-directional communication (Figure 1). This communication involves the innate and adaptive immune system as well (O’Riordan et al., 2025). For example, lipid absorption in mice involves communication between immune cells and enterocytes (intestinal epithelial cells) and modulated by gut microbiota (Burkhardt and Ecker, 2025). The importance of this communication is illustrated by the loss of the protein Aster-A in immune cells inhibiting lipid absorption and protecting mice against diet-induced obesity including adipose tissue inflammation and a fatty liver (Burkhardt and Ecker, 2025; Macadangdang et al., 2025). It is important to recognize that genomic elements like diversity-generating retroelements can hypermutate target genes in response to alteration in the gut environment (Paul and Mekalanos, 2025).

The vagus nerve, enteric nervous system, generation of neurotransmitters and steroid hormones like testosterone, and metabolites like short-chain fatty acids are an important part of this axis (Fulling et al., 2019; Alavian and Safaeian, 2025). For example, subdiaphragmatic vagotomy prevented detrimental effects of fecal implants from mice exposed to unpredictable chronic mild stress on reduced hippocampal neurogenesis, increased hippocampal neuroinflammation, and depressive-like behavior in recipient mice (Siopi et al., 2023). The role of the gut microbiome in behavioral, cognitive, and neuropathological measures is illustrated in Table 1.

**Table 1:** Role of the gut microbiome in behavioral, cognitive, and neuropathological measures.

Model	Association of gut microbiome with behavioral and/or cognitive performance measures	Association of the gut microbiome with hippocampal DNA methylation	Association of enhanced neuropathology by exposure of mice to the gut microbiome by co-housing with AD model mice	Causal role of gut microbiome on behavioral and/or cognitive phenotype following fecal implantation	References
Subdiaphragmatic vagotomy (SDV)	Association with depression-like phenotypes		SDV did not affect the reduction of tyrosine hydroxylase (TH) and dopamine transporter (DAT) in the striatum and increases in phosphorylated alpha synuclein in the colon after repeated MPTP administration	Prevention detrimental effects of fecal implants from mice exposed to unpredictable chronic mild stress on reduced hippocampal neurogenesis, increased hippocampal neuroinflammation, and depressive-like behavior	Siopi et al. (2023), Zhang et al. (2020), and Shan et al. (2022)
MPTP	Partly			Fecal implants from PD patients to MPTP-treated mice aggravated motor impairments, dopaminergic neurodegeneration, nigrostriatal glial activation and colonic inflammation, while fecal implants from healthy human controls improved the MPTP-caused effects	Torres et al. (2018) and Xie and et al. (2023)
PQ	Confirmed			Exposure of N27 dopaminergic cells to PQ induces histone H3 acetylation associated with decreased total histone deacetylase (HDAC) activity and HDAC4 and 7 protein expression levels and anacardic acid attenuates paraquat-induced caspase-3 enzyme activity, suppresses proteolytic activation and kinase activity of protein kinase C delta and PQ-induced cytotoxicity.	Feltzin et al. (2019), Anselmi et al. (2018), Chaklai et al. (2024), and Song et al. (2011)
Fecal implants of AD mouse models into germ-free mice	Confirmed		The oral and gut microbiota of AD patient partners resemble that of AD patients but differs from healthy controls, indicating the transmission of oral and gut microbiota and its impact on cognitive function.	Confirmed	Kundu et al. (2022b) and Zhang et al. (2023)
NL-G-F and NL-F APP KI AD mouse model		Confirmed	Mice transplanted with microbiota from conventionally bred 5XFAD mice show impaired memory performance, whereas fecal implants from mice housed in germ-free facility did not induce memory deficits in transplanted mice and 18 weeks of housing germ free-born animals in a conventional facility results in the reappearance of specific microbiota compositions in 5XFAD versus wild-type mice.	Confirmed	Okhovat et al. (2025) and Ismeurt-Walmsley et al. (2025)
3xTG AD mouse model	Gut microbiota modifications in 3xTg-AD mice anticipate in cognitive decline.		Confirmed		Chen et al. (2020), Bello-Medina and et al. (2021), and Li et al. (2023)
Germ-free mouse model	Impaired social avoidance and social novelty detection	Genome-wide DNA methylation analysis of hippocampal DNA identifies microbiome-associated differences in DNA methylation of 196 loci in total, 176 of which show conserved profiles between mother and offspring and single-cell transcriptional analysis reveals accompanying differences in expression of several differentially methylated genes within certain hippocampal cell clusters, and vascular expression of genes associated with bile acid transport.		Colonization of the gut at weaning	Desbonnet et al. (2013) and Gustafson et al. (2025)
Wild-type rodents	Modulated anxiety and depression	Confirmed	Confirmed	Transplantation of gut microbiota from healthy rats enhances cognitive function in male rats with traumatic brain injury caused by a gas explosion, through the modulation of gut microbiome composition and the improvement of both gut and brain barrier integrity via the gut-brain axis.	Krieger et al. (2022), Gustafson et al. (2025), Foster and McVey Neufeld (2013), Okhovat et al. (2025), Ismeurt-Walmsley et al. (2025), and Dong et al. (2024)
Wild-type rodents treated with antibiotics	Modulated anxiety and depression			Gut microbiota mediate cognitive impairment in young mice after multiple neonatal exposures to sevoflurane.	Dinan and Cryan (2017) and Liu et al. (2021)

Stimulation of the vagus nerve facilitates the extinction of fear learning (Burger et al., 2016). Based on this result, stimulation of the vagus nerve is being considered as therapeutic strategies in patients with post-traumatic stress disorder (PTSD; Genheimer et al., 2017; Bremner et al., 2021). As PTSD is a risk factor of Parkinson’s disease (PD; Weaver et al., 2024; Barer et al., 2022), cognitive decline (Prieto et al., 2023; Nilaweera et al., 2020), and Alzheimer’s disease (AD; Yaffe et al., 2010), targeting the gut-brain axis earlier in life might have long-term protective effects in these neurodegenerative conditions. This might be especially important in those with genetic factors of PD and/or AD.

Humans heterozygous for the glucocerebrosidase 1 (GBA) L444P Gaucher mutation have an increased PD risk (Malek et al., 2018; Migdalska-Richards and Schapira, 2016), and female and male mice heterozygous for the GBA mutation and expressing alpha synuclein with the A53T mutation and male mice with the A53T mutation, do not show extinction of fear memory (Bunnell et al., 2025). *PARK2*, which is involved in dopamine and associated with PD (Ming et al., 2020), is associated with PTSD in men (Nievergelt et al., 2019). The mutation enhanced the vulnerability of peripheral blood lymphocytes to paraquat (Ming et al., 2020), a herbicide linked to PD in most studies (Ball et al., 2019; Goldman, 2014; Forouzanfar et al., 2015; Wray, 2019; Tanner et al., 2011), especially following traumatic brain injury (Forouzanfar et al., 2015). The role of the gut-brain axis in the detrimental effects of paraquat is illustrated by the reduced sensitivity to this herbicide in germ-free mutant parkin flies (Feltzin et al., 2019) and that vagotomy prevents the development of PD symptoms and limits the appearance of misfolded aSyn in myenteric neurons in rats following co-administration of subthreshold doses of PQ and lectins (Anselmi et al., 2018). In addition, in mice heterozygous for the GBA mutation and expressing alpha synuclein with the A53T mutation or only expressing alpha synuclein with the A53T mutation mouse behavior linked to the taxonomic composition of the microbiome in ways that were influenced by PQ exposure (Chaklai et al., 2024). Consistent with the PQ exposure data, the effects of the neurotoxin 1-methyl-4-phenyl-1,2,3,6-tetrahydropyridine (MPTP) on cognitive performance may, at least in part, be mediated by the gut microbiome (Torres et al., 2018).

In the case of AD, our prior work revealed that transplanting stool samples from mouse models of AD into wildtype mice impaired mouse behavior and cognition in ways consistent with the disease (Kundu et al., 2022b). The behavior of gnotobiotic mice was assessed in a biosafety cabinet so that these efforts are not confounded by microbiome contamination. Transplanting stool collected from 6-month-old mice expressing human amyloid precursor protein (APP) containing dominant AD mutations NL-G-F (*App^NL-G-F^*) and *App^NLG-F^* mice crossed with human E4 targeted replacement mice (*App^NL-G-F/E4^*) is sufficient to induce behavioral phenotypes in 4–5 month-old germ-free C57BL/6 J mice 4 weeks following inoculation, as compared to wild-type transplantation controls but the host genotype modulated the pattern of induced behavioral phenotypes as compared to those seen in the genotype- and sex-matched donor mice (Kundu et al., 2022b). Insoluble Abeta40 levels were detected in *App^NL-G-F^* and *App^NL-G-F/E4^* recipient mice. Recipients of *App^NL-G-F^* donor mice carried cortical insoluble Ab40 levels that positively correlated with activity levels on the first and second day of open field testing. For recipient mice, the interaction between donor genotype and several behavioral scores linked to gut microbiome alpha-diversity (*p* < 0.05). Similarly, two behavioral performance scores predicted microbiome composition in recipient mice, but this association was dependent on the donor genotype (*p* < 0.05). Epigenetic changes in the hippocampus might be part the mechanisms mediating effects of the gut-brain axis in this model. The gut microbiome in *App^NL-G-F^* and *App^NL-F^* mice links to changes in the hippocampal epigenome, and that the nature of these associations differ as a function of genotype (Kundu et al., 2021). In the *App^NL-G-F^* mice, alterations in chromatin accessibility, gene expression, and DNA methylation are associated with early amyloidosis, and transcriptomic comparisons between *App^NL-G-F^* and wild-type mice revealed gene expression differences in pathways related to mitochondrial function and protein biosynthesis preceding amyloid plaque deposition (Okhovat et al., 2025). Although not assessed yet, it is conceivable that in mice, alterations in the gut microbiome precede amyloid plaque disposition as well. Once amyloid pathology is observed around 6 months of age, there is upregulation of immune and neuroinflammatory pathways. In addition to the hippocampus, DNA methylation differences during early and later stages of amyloid pathology are seen in blood that are associated with putative cis-regulatory elements in the mouse brain and were located near differentially expressed genes in the hippocampus. These regions were enriched in pathways associated with neuron development and synaptic processes (Okhovat et al., 2025). Thus, in addition to the gut microbiome and gut-brain axis, blood DNA methylation might serve as a biomarker for early detection of amyloid pathology.

In another pre-clinical AD model 3xTg, containing AD mutation APP_Swe_, presenilin 1 (PS1) with the M146V mutation, and tau with the P301L mutation, exposure of microbiota by co-housing with aged mutant mice, but not with aged wild-type mice, accelerated AD pathology in young 3xTg mice (Chen et al., 2020).

Apolipoprotein E (apoE) is involved in cholesterol metabolism and repair after injury. In humans, apoE exists in three major isoforms; E2, E3, and E4 (Goedert et al., 1994). Compared to E3, E4 increases while E3 decreased the risk to develop AD (Farrer et al., 1997). However, E2 (Johnson et al., 2015; Freeman et al., 2005; Kim et al., 2013) and E4 carriers both show increased risk to develop PTSD (Kimbrel et al., 2015; Lyons et al., 2013; Peterson et al., 2015). Consistent with these human data, young adult (4–5 or 3–6 month-old) group-housed E2 and E4 targeted replacement mice expressing human apoE under control of the murine apoE promoter, middle-aged group-housed E2, young singly-housed E4 and middle-aged singly housed E2 mice showed impaired extinction of contextual fear memory not seen in singly- or group-housed E3 mice (Johnson et al., 2015; Olsen et al., 2012; Saltonstall et al., 2025). *APOE* genotype, linked to both PTSD and AD, is associated with genotype-dependent gut microbiome profiles in humans and human apoE targeted replacement mice (Tran et al., 2019).

The role of the gut-brain axis in cognition might involve effects of the axis on the hippocampus (Salami and Soheili, 2022). Consistent with this role, germ-free mice show impaired social avoidance and social novelty detection when exposed to a chamber with or without a mouse or a familiar and novel mouse, respectively (Desbonnet et al., 2013). Colonization of the gut at weaning prevented the social avoidance but not the social novelty detection or transmission of social food preference (Desbonnet et al., 2013), highlighting that not all cognitive effects the gut-brain axis can be corrected later in life. The gut-brain axis also affects behavioral performance, including anxiety (Krieger et al., 2022) and depression (Foster and McVey Neufeld, 2013), which can be targeted with probiotics (Dinan and Cryan, 2017), and might in this way indirectly affect cognition.

Often studied in the context of the developmental origin of health and disease hypothesis (Gluckman and Hanson, 2004), many conditions developing later in life are hypothesized to have early foundations early in life. In this context, it is important to consider the role of the gut-brain axis in brain development, including cognitive development (Laue et al., 2022), and how this might ultimately result in cognitive injury in age-related neurodegenerative conditions.

### 1.2 The gut-brain axis and healthy cognitive aging in humans and animal models

The human gut microbiome diversifies with age, reflects healthy versus unhealthy aging, is associated with a healthy lipid profile, and predicts survival (Wilmanski et al., 2021). At midlife (mean age ± standard deviation: 55.2 ± 3.5 years of age), b diversity, a measure of gut microbial community composition, is associated with performance on cognitive tests, including the Montreal Cognitive Assessment (MoCA), Digit Symbol Substitution Test (DSST), Rey-Auditory Verbal Learning Test (RAVLT), Stroop, category fluency, and letter fluency tests (Meyer et al., 2022).

The gut-brain axis might be particularly important for Veterans. A common symptom among Veterans from different wars is gastrointestinal (GI) issues. They occur at a higher frequency in Gulf War (GW) Veterans and persist many years after the war (Dursa et al., 2016). GI problems are associated with neurological symptoms like cognitive dysfunction and fatigue (White et al., 2016). The percentage of GW Veterans affected with GI issues varies between cohorts and is estimated to be between 14 and 25% (Zhang et al., 2019). These studies suggest that the gut-brain axis might be disturbed in Veterans.

In cognitively healthy individuals (68–94 years old), the gut microbiome correlates of preclinical AD neuropathology (beta-amyloid and tau biomarkers) and inclusion of microbiome features associates with preclinical AD improves prediction of preclinical AD status (Ferriero et al., 2023). Consistent with these data, transplantation with fecal microbiota improves cognition in patients with cognitive decline and bacterial infection (Park et al., 2022), with various neurological conditions (Alaeddin et al., 2025), including mild cognitive impairment (Chen et al., 2023).

Transfer of aged donor microbiota into young mice accelerated age-associated inflammation in brain (Parker et al., 2022). Consistent with these mouse data, in rats, fecal transplants from 20 to 24 month-old aged male rats in antibiotic-treated 3-month-old male rats impaired performance in the delayed matching to position task, a working memory task, decreased dendritic spines in the medial prefrontal cortex and hippocampus, and reduced expression of brain-derived neurotrophic factor (BDNF), N-methyl-D-aspartate receptor NR1 subunit, and synaptophysin, and increased expression of advanced glycation end products (AGEs) and receptor for AGEs (RAGE; Li et al., 2020). Conversely, transference of microbiota from young mice into aged mice reversed the hallmarks of the aging brain (Parker et al., 2022). However, the presence of a gut microbiome and antibiotic treatment might complicate the interpretation of the data in this fecal transplant rat study. When 5–6 week-old germ-free mice received fecal transplants from 24-month-old mice, they showed increased hippocampal neurogenesis (Kundu et al., 2019).

### 1.3 The gut-brain axis and age-related cognitive injury in humans and animal models

Gut dysbiosis has been proposed as a hallmark of neurodegeneration (Molinero et al., 2023). For example, alterations in microbiome composition link to AD and impact AD-associated behaviors and brain pathologies. Greater dysbiosis of the gut microbiome is associated with worse cognitive function, including cognitive measures on the Mini Mental State Exam (MMSE) and Clinical Dementia Rating (sum of boxes) CDR (Son et al., 2025). This effect was partially mediated by greater brain age even when accounting for chronological age, sex, and education (Son et al., 2025). The response to supplements targeting the gut-brain axis might depend on the baseline gut microbiome condition. Probiotic supplementation improved cognitive performance in healthy older individuals, patients with mild cognitive impairment (MCI), and AD, but not in those with adequate physical activity meeting the exercise guidelines (Fekete et al., 2024; Sanborn et al., 2022).

Environmental challenges that are risk factor of developing AD pathology and AD (Graham et al., 1995) often affect the gut microbiome and it is conceivable that alterations in the gut microbiome following environmental challenges play a critical role in the detrimental effects on the brain. For example, traumatic brain (TBI), a risk factor for developing cognitive injury and AD pathology and AD (Jiang et al., 2013) and AD pathology in pre-clinical mouse models (Murai et al., 1998; Smith et al., 1998; Nakagawa et al., 1999) affect the gut microbiome in humans (McAllister, 2008; Rice et al., 2019) and in preclinical models (Taraskina and et al., 2022; Wang et al., 2021). In addition, post-TBI gut dysfunction exacerbates brain damage (Hanscom et al., 2021) and rates of neurological recovery following TBI associate with the gut microbiome (Yuan et al., 2021). The best-known genetic risk factor for poor outcome after TBI is E4 (Nathoo et al., 2003; Atherton et al., 2022) and apoE is also expressed at high abundance in the gut (El-Bahrawy et al., 2016).

Bile acids play a critical role in maintaining lipid, glucose, and energy homeostasis (Qi et al., 2015). Bile acid analyses in the gut can help in identifying the affected pathways involved in inflammation in the gut and brain. Dysbiosis of gut microbiota following TBI is associated with alterations of the bile acid profile (You et al., 2022). Bile acid synthesis and metabolism are altered in AD (Baloni et al., 2020), and the altered bile acid profile in AD is associated with cognitive impairments (Dehkordi et al., 2019). Bile acids are also involved in the protective effects of Xanthohumol (XN), a flavonoid produced by hops, against the detrimental effects of metabolic syndrome on cognitive function due to a high-fat diet (HFD; Miranda et al., 2018; Paraiso et al., 2020).

In addition to PD and AD, alterations in the gut microbiome are noted in other neurodegenerative disease as well. For example, in multiple sclerosis (MS), alterations in the gut microbiome linked to inflammation are seen (Kundu et al., 2022a; Altieri et al., 2023). Causality was shown by using monozygotic twins discordant for MS and fecal transplantation in germ-free mice and assess susceptibility to develop experimental autoimmune encephalomyelitis (EAE); MS-derived ileal microbiota induced EAE at higher rates and females were more susceptible to this than males (Yoon et al., 2025).

The gut microbiome is also linked with Amyotrophic Lateral Sclerosis (ALS) and progression of ALS (Fontdevila et al., 2024; Hertzberg et al., 2023; Sun and Zhang, 2024). The difference in the gut microbiome of ALS patients and controls correlates with plasma metabolites, especially lipids (Guo et al., 2024). Pre-clinical mouse models reveal GI symptoms before motor symptoms and alterations in the gut microbiome (for a review, see Martin et al., 2022).

**Figure 2:**
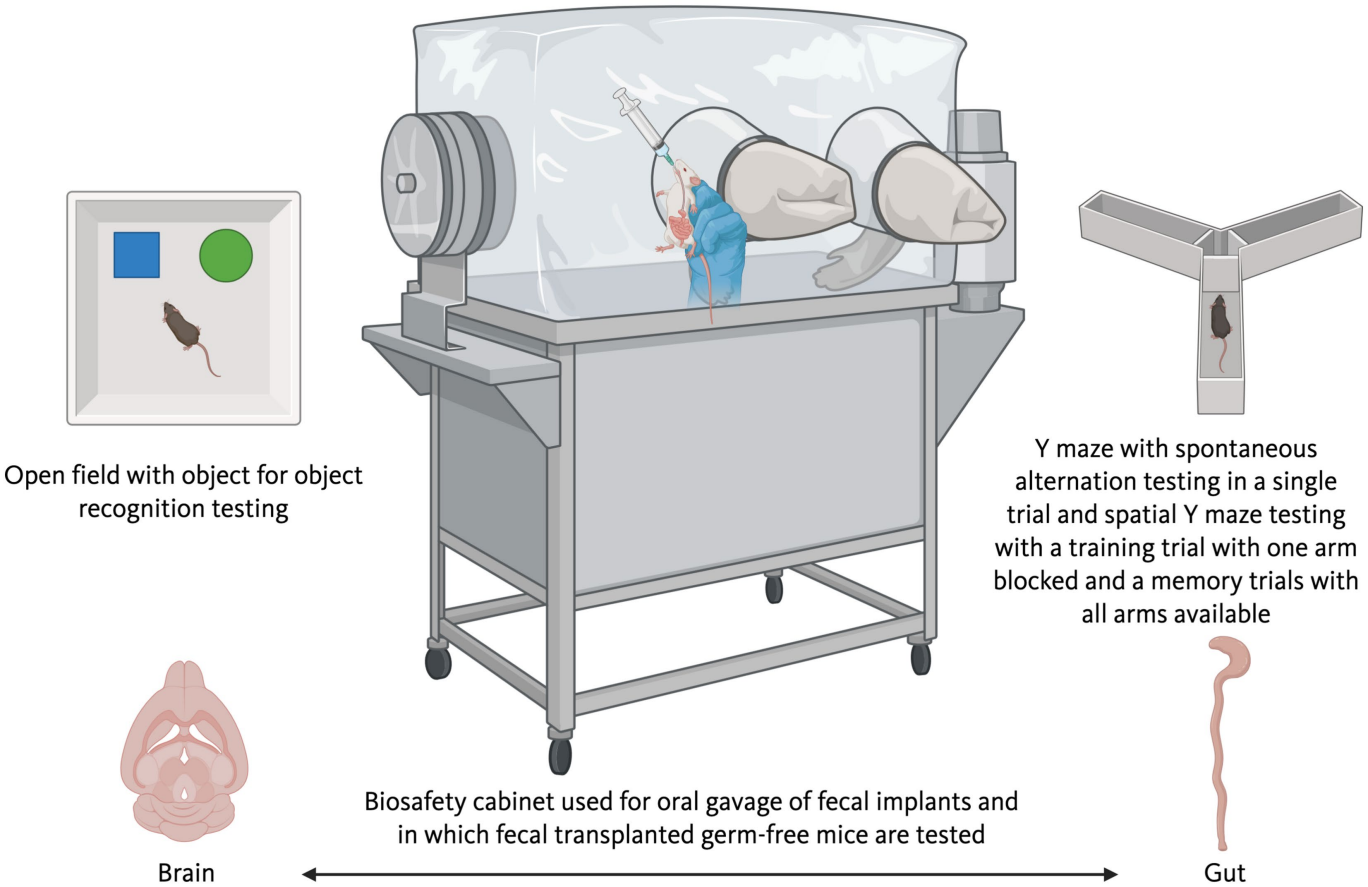
To determine causality and to assess whether alterations in the gut microbiome are sufficient to induce cognitive and neurodegenerative phenotypes, human stool or fecal matter of animals can be administered in germ-free mice via oral gavage in a biosafety cabinet. Following colonization, the mice can be cognitive tested in a biosafety cabinet, to minimize cross-contamination of the gut microbiome, and subsequently the brain (lower left image) and gut (lower right image) can be analyzed to assess the effects of the fecal implantation, to determine the relationship of the gut microbiome in the donor and recipient, and to assess the relationship between the gut microbiome and cognitive phenotypes in the recipient mice. There are limitations to testing inside a biosafety cabinet and tests like the open field, object recognition test (top left image), and Y maze (top right image) are suitable based on their limited footprint.

**Figure 3:**
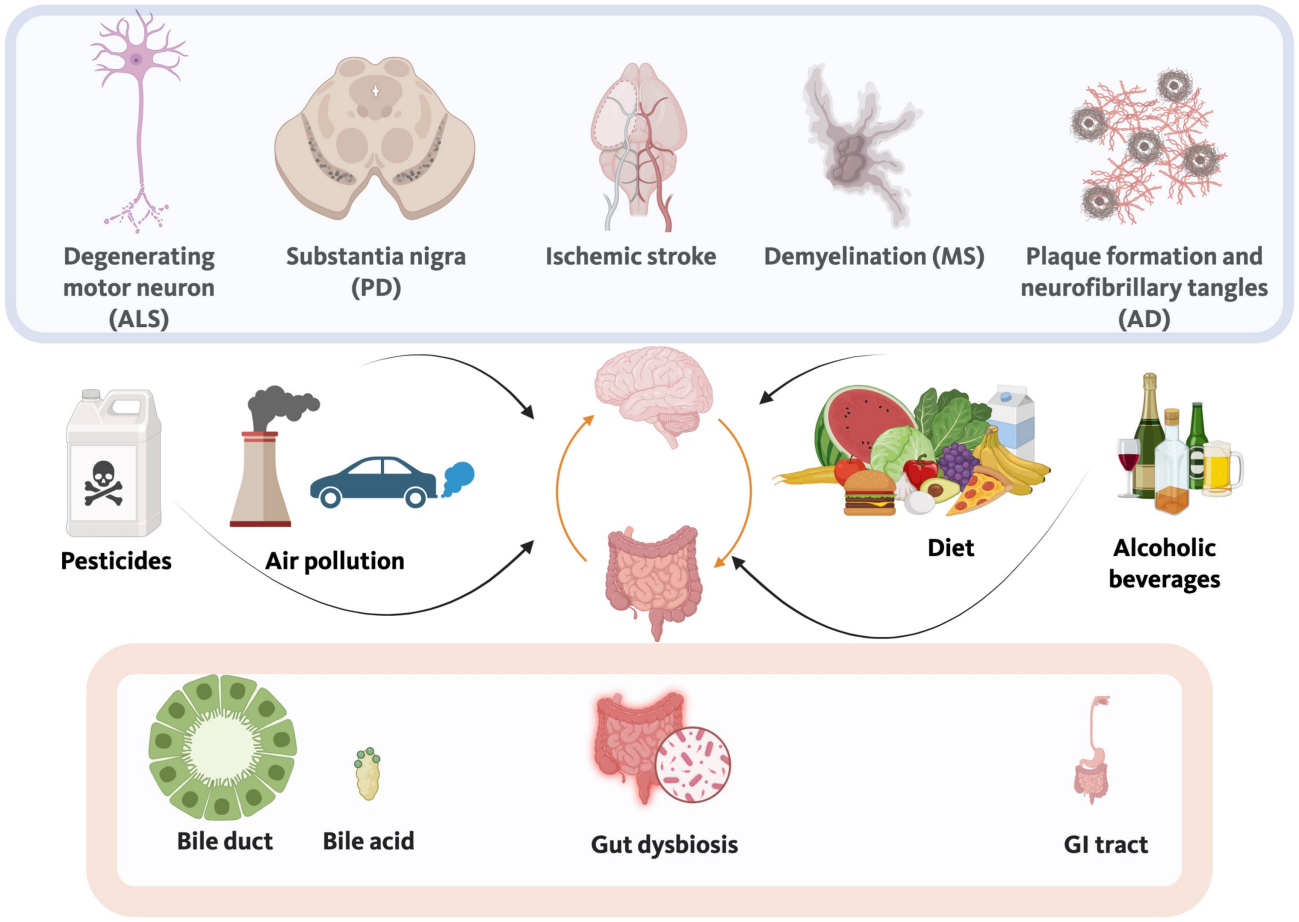
Environmental factors, including diet, alcohol use, pesticide use and exposure, and air pollution can affect the gut-brain axis. A role for alterations in the gut microbiome and the gut-brain axis has been implicated in various neurodegenerative conditions, including AD, PD, MS, ALS, and ischemic stroke. In the gut, inflammation and dysbiosis and alterations in bile acid synthesis and metabolism are associated with cognitive injury in neurodegenerative conditions like AD.

Alterations in the gut microbiome are also implicated in vascular cognitive impairment and vascular dementia (Yang et al., 2024; Li et al., 2018). Alteration in the gut microbiome are also seen in the occurrence and development of ischemic stroke (Wang et al., 2022; Roth et al., 2024) and hemorrhagic stroke (Shen et al., 2023; Li et al., 2025; Ye et al., 2025; Figures 2, 3).

These studies show that there might be a general theme in various neurodegenerative disorders with alterations in the gut microbiome and associated inflammation in the gut driving detrimental changes in the brain via the gut-brain axis. As more disease-specific data sets become available, it will be important to distinguish disease-unique versus overlapping microbiome signatures that are associated with brain disease-specific versus brain overlapping disease symptoms. Increased understanding of this distinction will provide more mechanistic insights in the different neurodegenerative conditions. From a therapeutic perspective, the overlapping microbiome signatures might allow the development of strategies to reduce brain injury in various neurodegenerative conditions. Still, the disease-specific microbiome signatures will be important to note as well as they might need to be targeted as well for optimal brain function in the patients.

Similarly, a healthy gut microbiome might delay, reduce, prevent, and/or successfully treat these neurodegenerative conditions (Ma et al., 2024; Munir et al., 2024; Chui et al., 2024).

Based on the promise of targeting the gut microbiome and gut-brain axis as therapeutic target in neurodegenerative conditions, there are two current research gaps to consider: (1) novel ways to modulate the gut microbiome to improve cognitive function; and (2) using the gut microbiome, cognitive scores, and diet questionnaire-derived scores to develop novel interventions to optimize cognitive aging and cognitive injury in neurodegenerative conditions. Diet has critical effects on the gut microbiome and is a modifiable factor to reduce brain injury and improve brain function. Both research gaps are briefly discussed below.

### 1.4 Current research gap: a methodological framework for diet-microbiome-cognition research

**Figure 4:**
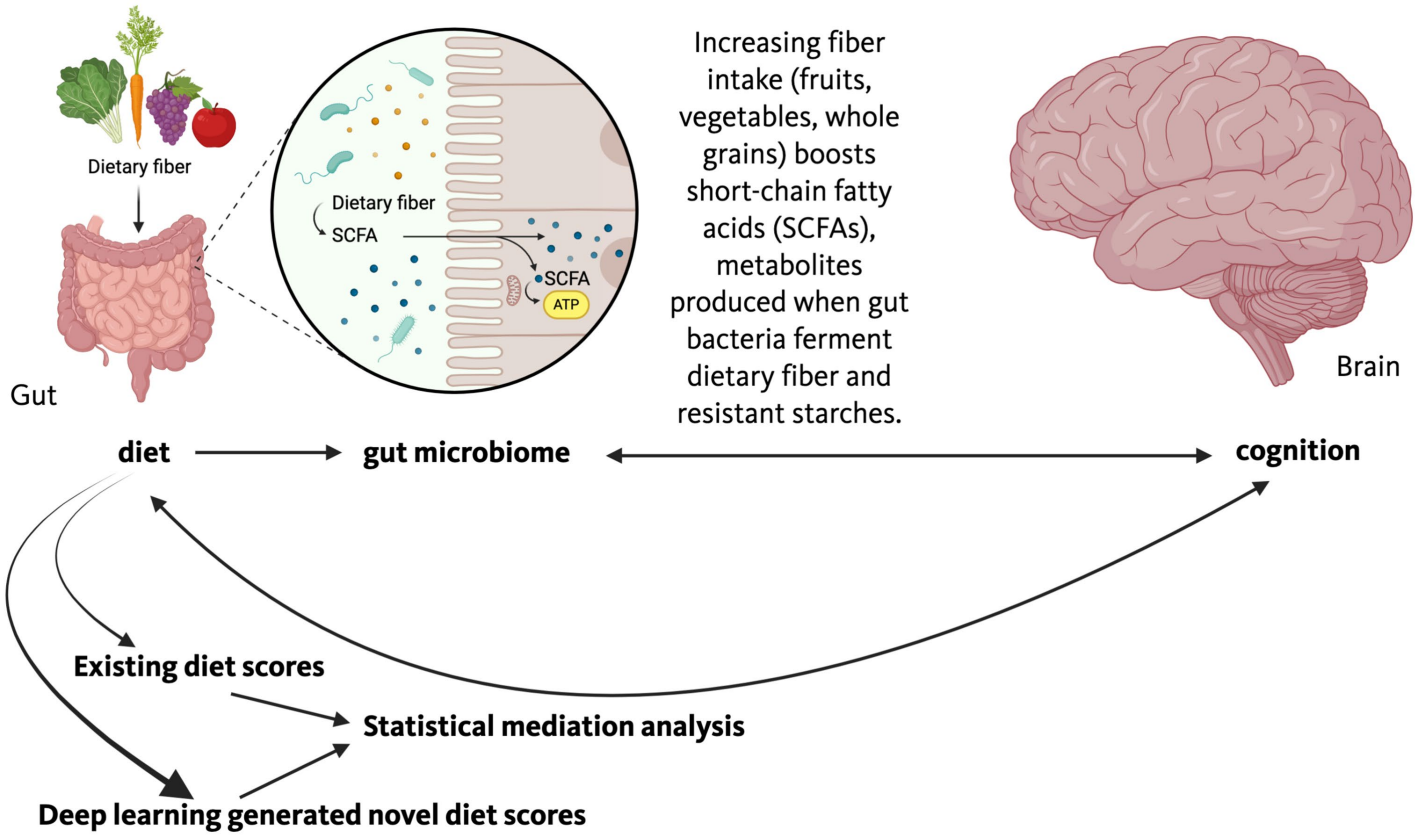
Schematic workflow for diet-microbiome-cognition research. Dietary intake data, typically collected via food frequency questionnaires, serve as the starting point for two complementary analytical tracks. In the first, existing diet scores (e.g., MEDAS, MIND, HEI; Table 2) are calculated and tested for associations with cognitive outcomes. In the second, supervised approaches such as reduced rank regression, regularized regression, or deep learning derive novel diet scores optimized to explain variance in cognitive performance (see Table 3 for method selection guidance). Statistical mediation analysis decomposes the diet–cognition relationship into direct effects and indirect effects operating through the gut microbiome, with microbiome composition and microbial metabolite profiles as candidate mediators. Validation requires internal cross-validation, external replication in independent cohorts, and biological validation against mechanistic intermediates (e.g., short-chain fatty acids, bile acids, inflammatory markers). For details, see Sections 1.5.1–1.5.6.

Based on the promise of targeting the gut microbiome and gut-brain axis as therapeutic targets in neurological conditions, there are two current research gaps to consider: (1) novel ways to modulate the gut microbiome to improve cognitive function, and (2) using the gut microbiome, cognitive scores, and diet questionnaire-derived scores to develop novel interventions to optimize cognitive aging and reduce cognitive injury in neurodegenerative conditions (Cryan et al., 2019). For a Schematic workflow for diet-microbiome-cognition research (see Figure 4). Diet surveys are most often used, but other diet measurements like diet journals and deep learning tools to impute diet composition based on photos of meals might be considered as well. While empirical tests afford the strongest insight into cause-and-effect relationships, they are often challenging to implement in an epidemiological context, and causality studies involving germ-free mice are expensive and require specialized resources. In human studies, assessing the effects of modulation of the gut microbiome on cognition through dietary interventions is labor-intensive and costly. Therefore, there is increasing interest in using diet questionnaire-derived scores that can explain variance in cognitive performance and guide recommendations for modulation of the gut microbiome in patients with neurodegenerative conditions and those at high risk of developing them. Realizing this potential requires careful attention to causal inference frameworks, analytical approaches, sample size considerations, validation strategies, and individual heterogeneity.

Several established approaches provide starting points for diet-cognition research. One option is to calculate a prudent diet score based on a full food frequency questionnaire (or a short questionnaire derived from it) and assess relationships with relevant biomarkers (Robinson et al., 2017). Another option is to use diet questionnaire data to calculate Mediterranean diet adherence indices such as the Alternate Mediterranean Diet Score (aMed) as operationalized in prior work on diet quality indices (Fung et al., 2005) or the 14-item Mediterranean Diet Adherence Screener (MEDAS), which was validated in the PREDIMED study (Schröder et al., 2011; Table 2). An advantage of this approach is that Mediterranean-style dietary patterns have been associated with better cognitive outcomes and reduced risk of neurodegenerative disease in multiple lines of evidence, including both cohort studies and randomized trial evidence for cognitive endpoints (Scarmeas et al., 2006; Gardener and Caunca, 2018; Valls-Pedret et al., 2015). In addition, Mediterranean-style dietary patterns have been linked to favorable gut microbiota features in human studies and reviews (Barber et al., 2023). Beyond Mediterranean indices, brain-health–focused and guideline-based diet quality scores derived from questionnaire data, such as the MIND diet score (Morris et al., 2015a; Morris et al., 2015b) and the Healthy Eating Index (HEI; Krebs-Smith et al., 2018), have also been evaluated in cognitive aging cohorts (Haring et al., 2016). However, diet scores affecting cognition may differ based on disease condition, genotype, and environmental context. A data-driven approach using the supervised methods described above could identify disease-specific or population-specific dietary patterns that outperform generic scores. The dietary components identified through such analyses could subsequently inform dietary recommendations to improve cognition in humans and guide diet intervention studies in preclinical models to assess the pathways in the gut, brain, and microbiome that drive these beneficial effects.

**Table 2:** Diet scores based on food frequency questionnaires.

Diet score	Characteristics and differences	Impacts	References
MEDAS	No specific requirements for berries or leafy greens, allows for moderate dairy intake, and less restrictive of sodium compared to MIND.	Reduced dementia risk. Reduced cardiovascular risk, BMI, and improvement in metabolic markers.	Liu et al. (2025), Gregory et al. (2022), Youn et al. (2025), Fox et al. (2022), and Devranis et al. (2023)
MIND	Green leafy vegetables, berries; more strict on reducing intake of butter, margarine, and fast and fried foods than MEDAS.	Reduced dementia risk. Improved working memory, attention, and verbal recognition.	Liu et al. (2025), Fox et al. (2022), Devranis et al. (2023), Chen et al. (2024), Yu and et al. (2025), and Nishi and et al. (2021)
HEI	Measure of diet quality based on dietary guidelines for Americans. General metric of compliance with US national guidelines and not specific dietary patterns as MIND and MEDAS.	Reduced dementia risk.	Youn et al. (2025), Hu et al. (2025), and Ayala-Garcia et al. (2024)

#### 1.4.1 Statistical mediation for causal inference

The conceptual model underlying diet-microbiome-cognition relationships posits that dietary intake influences cognitive outcomes at least partially through modulation of the gut microbiome. Statistical mediation analysis provides a formal framework for testing this hypothesis by decomposing the total effect of diet on cognition into direct effects and indirect effects operating through the microbiome (VanderWeele, 2016). However, several methodological challenges must be addressed. First, causal interpretation of mediation effects requires strong assumptions, including (among others) no unmeasured confounding of the diet–microbiome, microbiome–cognition, and diet–cognition relationships, and correct specification of temporal ordering (VanderWeele, 2016). In cross-sectional studies where diet, microbiome, and cognition are measured simultaneously, these assumptions are difficult to justify, and cross-sectional mediation estimates can be substantially biased relative to longitudinal processes (Maxwell and Cole, 2007). Longitudinal designs with repeated measures of all three domains strengthen causal inference by establishing temporal precedence (VanderWeele, 2016; Maxwell and Cole, 2007).

Second, the gut microbiome is inherently high-dimensional, comprising hundreds to thousands of taxa or functional features. Traditional mediation approaches designed for single mediators do not readily extend to this setting. Recent methodological advances address this gap. Information-based approaches for high-dimensional mediation analysis can identify microbial features that may mediate exposure–outcome associations while controlling error rates in high-dimensional settings (Carter et al., 2020). In addition, because sequencing-based microbiome profiles are compositional (relative abundances constrained by a sample-specific total), mediation approaches that explicitly accommodate compositional structure can reduce artifacts that arise when naive regression is applied to relative-abundance features (Tsilimigras and Fodor, 2016; Gloor et al., 2017). Recent causal mediation frameworks have been developed specifically for high-dimensional and compositional microbiome mediators (Wang et al., 2020; Sohn and Li, 2022).

Third, the microbiome may act as a mediator for some dietary components but as a moderator (effect modifier) for others. For example, baseline microbiome composition may determine whether an individual responds to a particular dietary intervention (a moderation effect), while also transmitting the effects of habitual diet on long-term cognitive trajectories (a mediation effect). Analytic frameworks that accommodate both mediation and moderation, such as moderated mediation models, may better capture this complexity (Preacher et al., 2007).

Despite these advances, statistical mediation with observational data remains inferential rather than definitive (VanderWeele, 2016). Triangulation across complementary study designs (e.g., observational cohorts, mechanistic animal experiments, and human interventions) can strengthen causal inference when different approaches with distinct bias structures support the same mechanistic conclusion (Lawlor et al., 2016).

#### 1.4.2 Analytical approaches for deriving diet scores

**Figure 5:**
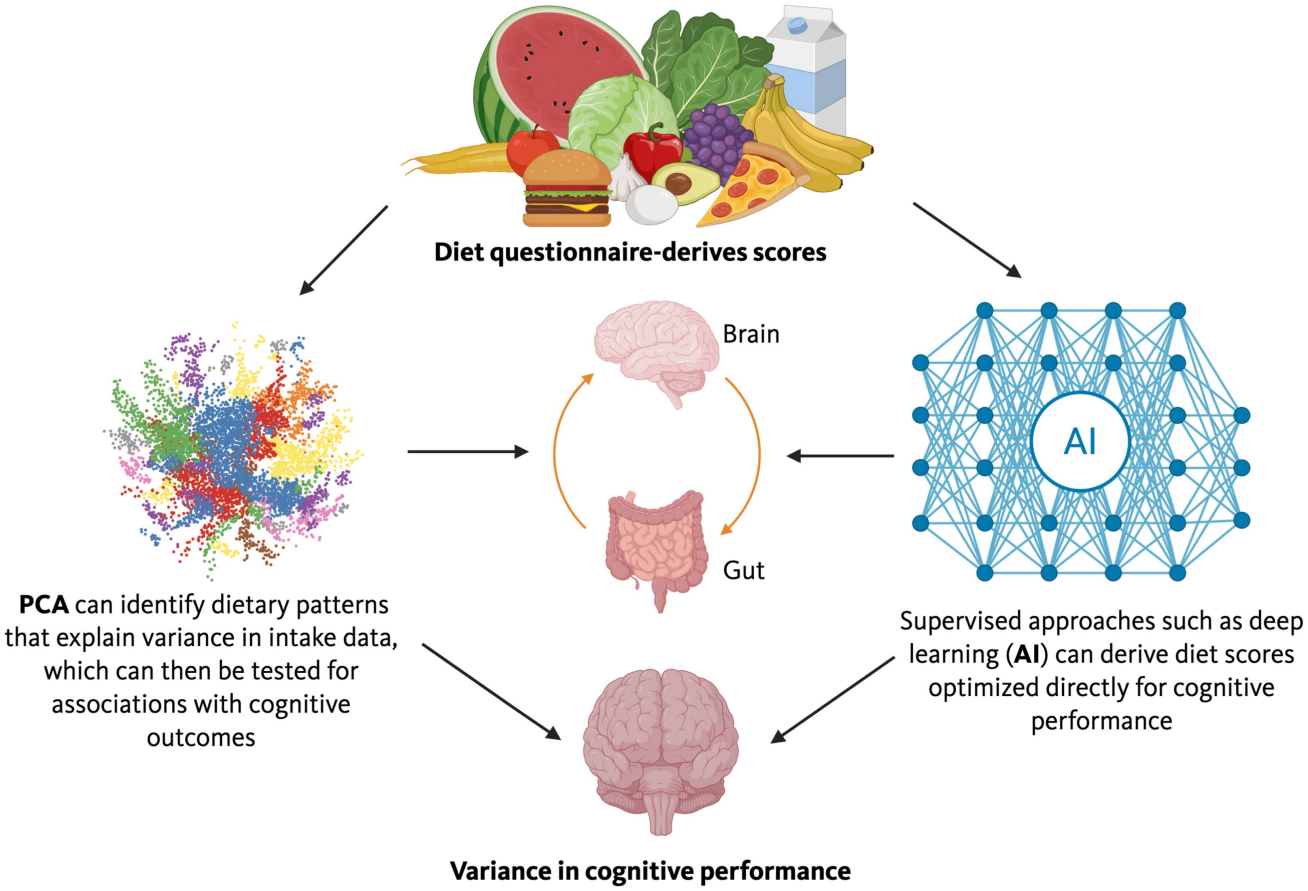
Diet questionnaires contain many measures that are often unwieldy to use unless reduced to diet scores. The left pathway illustrates an unsupervised approach: principal components analysis (PCA) identifies dietary patterns that explain variance in intake data, which can then be tested for associations with cognitive outcomes. The right pathway illustrates supervised approaches: methods such as reduced rank regression, partial least squares, regularized regression (LASSO/elastic net), or deep learning derive diet scores optimized directly for cognitive performance. Choice of method depends on the inferential goal, sample size, and expected complexity of diet–cognition relationships (Table 3). The resulting scores can inform dietary recommendations and guide diet intervention trials in both human cohorts and preclinical models.

The choice of statistical method for deriving diet scores should align with the inferential goal. Principal components analysis (PCA), while commonly used in nutritional epidemiology to derive dietary patterns (Figure 5), is an unsupervised technique that identifies patterns explaining variance in dietary intake data rather than patterns optimized for prediction of health outcomes (Hu, 2002; Hoffmann et al., 2004). When the goal is to identify dietary patterns that maximize explained variance in cognitive performance (or other targeted outcomes), supervised methods are often more appropriate (Hoffmann et al., 2004).

Reduced Rank Regression (RRR) derives dietary patterns that explain maximum variance in a set of response variables and has been widely used in nutritional epidemiology when the goal is to identify dietary patterns most strongly related to selected intermediate markers or outcomes (Hoffmann et al., 2004). Partial Least Squares (PLS) regression offers a related framework that constructs latent components to optimize prediction of response variables (Hoffmann et al., 2004). For high-dimensional diet data with many correlated food items, regularized regression approaches such as LASSO or elastic net can simultaneously perform variable selection and prediction, identifying dietary components most strongly associated with cognitive outcomes (Tibshirani, 1996; Zou and Hastie, 2005).

Deep learning approaches offer additional flexibility, particularly for capturing non-linear relationships and higher-order interactions between dietary features and cognition (LeCun et al., 2015; Ching et al., 2018). However, interpretability remains a challenge. While a deep learning model may achieve strong predictive accuracy, understanding which dietary components drive the prediction is less straightforward than with traditional regression approaches (Guidotti et al., 2018). Recent advances in explainable AI (XAI), including SHAP-style feature attribution and attention-based model families (and their associated interpretation strategies), can partially address this limitation by estimating feature importance and identifying influential input regions or features (Guidotti et al., 2018; Lundberg et al., 2020; Niu et al., 2021).

To aid method selection, Table 3 summarizes the key trade-offs among these approaches. In general, PCA is appropriate when the goal is to characterize dietary patterns without reference to a specific outcome; RRR and PLS are preferred when pre-specified intermediate response variables (e.g., biomarkers) are available; regularized regression is well-suited to direct prediction of cognitive outcomes with simultaneous variable selection; and deep learning offers flexibility for non-linear relationships at the cost of interpretability and increased data requirements. These methods are implemented in freely available software, including FactoMineR and sklearn.decomposition for PCA, pls and mixOmics for PLS, glmnet and sklearn.linear_model for regularized regression, and keras/torch for deep learning, with *post-hoc* interpretability supported by SHAP-based tools (fastshap, shap; Devranis et al., 2023; Chen et al., 2024; Yu and et al., 2025). For high-dimensional and compositional microbiome mediation analysis, specialized packages such as ccmm and SparseMCMM in R accommodate the constraints of sequencing-derived relative abundance data (Schröder et al., 2011; Valls-Pedret et al., 2015; Barber et al., 2023). Appropriate sample sizes for these approaches depend on the effect size of interest, the ratio of candidate predictors to observations, the correlation structure of dietary variables, and the validation strategy employed (Babyak, 2004; Steyerberg et al., 2001).

**Table 3:** Decision guide for selecting a diet score derivation method.

Consideration	PCA	RRR/PLS	LASSO/elastic net	Deep learning
Inferential goal	Describe dietary patterns	Predict pre-specified intermediate outcomes	Predict outcome with variable selection	Capture non-linear and interaction effects
Requires pre-specified response variables?	No	Yes (RRR); optional (PLS)	No	No
Handles high predictor-to-sample ratio?	Yes	Requires careful regularization when p approaches n	Yes	Requires relatively larger samples
Captures non-linear relationships?	No	No	No (without feature engineering)	Yes
Interpretability	High (loadings)	High (loadings)	High (coefficients)	Low; requires *post-hoc* explainability methods
Primary limitation	Patterns not optimized for outcomes	Requires selection of response variables	Assumes linearity	Overfitting risk; interpretability

#### 1.4.3 Sample size considerations

The sample size requirements for developing robust diet–cognition scores vary substantially by method, the number of candidate dietary predictors (and how they are grouped), and the dimensionality of the outcome space. Across modeling families, a central risk is overfitting when model flexibility is high relative to the available sample size, which can yield overly optimistic apparent performance and unstable feature selection (Babyak, 2004; Steyerberg et al., 2001). This concern is particularly acute for high-dimensional predictors, complex interaction structures, and highly flexible models such as deep neural networks (LeCun et al., 2015; Ching et al., 2018).

Given that individual studies with comprehensive diet, cognition, and microbiome data are often limited in size, pooled analyses across cohorts offer a promising path forward. While challenging, harmonization of variables across studies can enable development and validation of diet scores with greater statistical power and generalizability (Fortier et al., 2011). Federated learning approaches, which train models across distributed datasets without centralizing raw data, may facilitate such multi-cohort efforts while addressing data-sharing constraints (Rieke et al., 2020).

#### 1.4.4 Validation strategy

Rigorous validation is essential before diet-derived scores can inform clinical recommendations. Internal validation through k-fold cross-validation or bootstrap resampling provides initial estimates of model performance and helps quantify and reduce overfitting (Steyerberg et al., 2001). However, external validation in independent cohorts is critical for establishing generalizability across populations that may differ in demographic composition, dietary culture, and genetic background (Collins et al., 2015).

Prospective validation, which demonstrates that baseline diet scores predict future cognitive trajectories, provides strong evidence for clinical utility (Collins et al., 2015). Ideally, a diet–cognition score would be validated against longitudinal cognitive decline, incident mild cognitive impairment, or dementia diagnosis in cohort studies with extended follow-up.

Biological validation offers complementary evidence by testing whether diet scores associate with putative mechanistic intermediates. Scores designed to optimize cognitive outcomes via the gut–brain axis should correlate with microbiome composition and microbial metabolite profiles (e.g., short-chain fatty acids and bile acids), as well as systemic inflammatory markers and gut barrier integrity measures (Dalile et al., 2019; Monteiro-Cardoso et al., 2021; Di Vincenzo et al., 2024). Such associations would strengthen confidence that the diet score captures biologically meaningful variation rather than confounded associations.

#### 1.4.5 Addressing individual heterogeneity

A fundamental challenge in translating population-level diet–cognition associations to individual recommendations is the substantial inter-individual variability in gut microbiome composition and dietary response. The same dietary intervention can produce markedly different microbiome shifts across individuals, influenced by baseline microbiome composition, host genetics, medication use, and environmental factors.

Several strategies can address this heterogeneity. First, stratified analyses by enterotype or baseline microbiome features may reveal diet–cognition associations that are masked in unstratified analyses (Aramugam et al., 2011). For example, individuals with Prevotella-dominant versus Bacteroides-dominant microbiomes may respond differently to fiber-rich dietary patterns (Kovatcheva-Datchary et al., 2015) or nutritional interventions (Jamieson et al., 2024). Second, incorporating microbiome features directly into predictive models, rather than treating diet and microbiome as separate domains, may improve prediction by capturing diet–microbiome interactions and baseline-dependent responses (Zeevi et al., 2015). Third, the concept of “responders” versus “non-responders” to dietary interventions, which is well-established in the personalized postprandial glycemic response literature and more recently in glucosinolate metabolism, may generalize to other endpoints and motivates identifying baseline predictors of response (Zeevi et al., 2015; Bouranis et al., 2021).

Ultimately, the field may benefit from a precision nutrition framework in which diet recommendations are tailored based on an individual’s microbiome profile, genetic risk factors (e.g., *APOE* genotype), and baseline cognitive status. Such an approach would require development of decision algorithms integrating multiple data streams, validated in interventional trials powered to detect heterogeneous treatment effects (Collins et al., 2015; Zeevi et al., 2015).

### 1.5 Future perspectives; novel ways to modulate the gut microbiome to improve cognitive function

In addition to pre- and pro-biotics, diet and food supplements, and fecal transplants, using engineered microbial strains to improve cognitive health and treat cognitive injury are promising therapeutic strategies. To avoid survival of these engineered microbial strains outside the person treated and to avoid transfer of genetic material to other microbe and genetic instability, genetic containment is critical. For ethical consideration, please see Ahmed (2023). For regulatory and analytical challenges in the development and manufacturing of live biotherapeutic product development (M.T.I. Group and Barberio, 2024). An example of an effort along these lines is the recently reported “Sequence enTAngLEd Multi lAyered geneTic buffering” (STALEMATE; Foo et al., 2025). This system contains a dual-layered failsafe biocontainment strategy that entangles genetic sequences to create pseudo-essentiality and buffer against mutations. For example, the colicin E9 immunity protein (Im9) with a thermoregulated meganuclease (TSM) was entangled by overlapping the reading frames. Mutations that disrupt this entanglement simultaneously inactivate both biocontainment layers, resulting in cell death by the ColE9 nuclease and the elimination of escape mutants. By lengthening the entangled region, refining ColE9 expression, and optimizing the TSM sequence against IS*911* insertion, escape rates below 10^−10^ as compared to rates of 10^−5^ with the nonentangled TSM can be achieved (Foo et al., 2025). Plasmids generated using this system were contained for 7 days in the mouse gastrointestinal tract with nearly undetectable escape rates upon excretion.

### 1.6 Summary

Statistical mediation offers an inferential solution and have recently been applied to microbiome studies (Carter et al., 2020). This is especially important as while empirical tests afford the strongest insight into cause-and-effect relationships, they are often challenging to implement in an epidemiological context and causality studies involving germ-free mice are expensive and require special resources. In human studies, assessing the effects of modulation of the gut microbiome on cognition by using special diets, diet restriction/adjustment, or diet supplements, is very involved as well. Therefore, there is increasing interest in using diet questionnaire-derived scores that can explain most of the variance in cognitive performance and that can in turn be used to guide recommendations for modulation of the gut microbiome in patients with neurodegenerative conditions and those at high risk of developing them. One option is to calculate the prudent diet score based on a full food frequency questionnaire using a principal components analysis or a diet score based on a short form of this questionnaire and assess their relationships with plasma vitamin C, high-density lipid particles (HDL), and serum triglycerides as reported (Robinson et al., 2017). Another option is to use diet questionnaire data to calculate the Alternative Mediterranean Diet Score as described by Fung et al. (2006). An advantage of this option is that in most studies the Mediterranean diet was shown to enhance cognition and reduce the risk to develop neurodegenerative conditions, including AD and PD (Gardener and Caunca, 2018; Liu et al., 2025; Picone et al., 2024). In addition, evidence supports that a Mediterranean diet has beneficial effects on the gut microbiome (Barber et al., 2023; Perrone and D’Angelo, 2025; Khavandegar and et al., 2024). As diet scores affecting cognition might be different based on disease condition, genotype, and environmental condition, a third option is to use deep learning to determine the diet score that can explain most of the variance in cognition in each disease condition and data set. The parts of the diet the diet score is based on might then subsequently be used to develop dietary recommendations to improve cognition in humans and to develop diet intervention in preclinical models to assess the pathways in the gut, brain, and alterations in the gut microbiome that might drive these beneficial effects (Figure 5). A limitation of this approach is that this kind of analysis requires sufficient variance in diets of individual study participants and variance of their cognitive scores. In some cases, the sample size might be a limitation for this kind of analysis. Below, we indicate five considerations regarding this approach going forwards.

What to consider going forwards:

- Optimize the study design to allow sufficient variance in diet and cognitive scores.
- Compare established diet scores and deep-learning derived diet scores to explore which diet score drives enhanced cognitive performance.
- Consider dietary advice to improve cognition based on the generated data.
- Consider genetic factors and personalized medicine approaches for recommendations to improve cognition in healthy people and patients.
- Validate the data derived from human studies in environmentally controlled animal studies.
